# Efficacy and Implementation of Stress-Reduction Interventions for Underserved Families of Autistic Preschoolers Across In-Person and Virtual Modalities

**DOI:** 10.1007/s12671-023-02250-0

**Published:** 2023-11-14

**Authors:** Rachel M. Fenning, Cameron L. Neece, Catherine M. Sanner, Holly E. R. Morrell

**Affiliations:** 1 Department of Psychological Science and The Claremont Autism Center, Claremont McKenna College, 850 Columbia Avenue, Seaman Hall 103, Claremont, CA 91711, USA; 2 Department of Psychology, Loma Linda University, 11130 Anderson Street, Suite 119, Loma Linda, CA 92354, USA

**Keywords:** Mindfulness, Psychoeducation, Randomized controlled trial, Autism spectrum disorder, Parenting stress

## Abstract

**Objectives:**

Parents of autistic children experience elevated stress relative to parents of children with neurotypical development and children with other intellectual and developmental disabilities. Adverse effects of parenting stress on parent, child, and family functioning may be especially heightened for marginalized families. We conducted a randomized controlled trial that demonstrated the efficacy of Mindfulness-Based Stress Reduction (MBSR) relative to psychoeducational support (PE) for reducing stress in diverse and underserved parents of autistic preschoolers. This paper presents implementation data, and examines efficacy across in-person and virtual intervention modalities.

**Method:**

Primary caregivers (*n* = 117; 91% female, 51% Latinx, 44% income < US $50,000) of 3- to 5-year-old autistic children (80% male, 68% with intellectual disability) were randomly assigned to MBSR (*n* = 59, 46% virtual) or PE (*n* = 58, 41% virtual). Assessments were conducted at baseline, immediately post-intervention, and at 6 and 12 months post-intervention.

**Results:**

Both MBSR and PE demonstrated strong feasibility, acceptability, and utility for our diverse families. Comparable efficacy was observed across modalities. However, attendance was significantly better for virtual groups than for in-person groups. Parents participating in virtual MBSR also reported less difficulty completing homework and utilizing learned skills in everyday life than did in-person MBSR participants.

**Conclusions:**

MBSR and PE appear feasible, acceptable, and efficacious for diverse and underserved parents of young autistic children. Preliminary evidence of comparable efficacy across virtual and in-person modalities indicates the potential to expand access to vital stress-reduction interventions through use of telehealth technology.

**Preregistration:**

ClinicalTrials.gov Identifier: NCT03459625.

Many parents of autistic children find great happiness and meaning in the parenting role while also experiencing considerable challenges, including significant parenting stress (Myers et al., 2009). Parenting stress is commonly conceptualized as the stress or tension that arises when the perceived demands or requirements of the parenting role exceed parental resources (e.g., Abidin, 1992). Parents of autistic children report greater parenting stress than do parents of children with neurotypical development and parents of children with other intellectual and developmental disabilities (IDD) and chronic conditions (e.g., Barroso et al., 2018; Estes et al., 2009; Dabrowska & Pisula, 2010; see also Hayes & Watson, 2013). Numerous studies have linked elevated parenting stress with a host of adverse outcomes in families of autistic children, including poorer parent mental health (e.g., Enea & Rusu, 2020; Weitlauf et al., 2014), harsher parenting behaviors (Shawler & Sullivan, 2017), and transactional escalations in child behavior problems over time (Lecavalier et al., 2006; Osborne & Reed, 2009; Rodriguez et al., 2019; Zaidman-Zait et al., 2014, 2017). Parenting stress may also impede the uptake and effectiveness of early intervention (Osborne et al., 2008), underscoring the urgency of reducing strain in parents of young autistic children.

Multiple factors may contribute to heightened parenting stress in these families, including parental coping styles, appraisal processes, and mental health problems as well as children’s autism-related symptoms and co-occurring conditions, particularly externalizing behavior problems (Enea & Rusu, 2020; Kiami & Goodgold, 2017; Miranda et al., 2019; Rodriguez et al., 2019; Weitlauf et al., 2014; Zaidman-Zait et al., 2014; Zaidman-Zait et al., 2017; see also Fenning & Butter, 2019 and Karst & Van Hecke, 2012). External stressors tied to financial demands, resource needs, and service access, navigation, and receipt are often uniquely pronounced and persistent in this population (Grindle et al., 2009; Hastings & Beck, 2004; Karst & Van Hecke, 2012; Kiami & Goodgold, 2017). Moreover, parents of young autistic children are frequently still adjusting to the diagnosis itself (Wachtel & Carter, 2008). For families from underserved and marginalized communities, marked service disparities further exacerbate parenting stress (e.g., Angell et al., 2018; Iadarola et al., 2019; Magaña et al., 2012; Rivera-Figueroa et al., 2022; Wallace-Watkin et al., 2023; Williams et al., 2021). High levels of mental health and disability-related stigma may especially compound strain for Latinx parents (Rivera-Figueroa et al., 2022; Zuckerman et al., 2014).

Despite evidence of pervasive parenting stress and long-lasting negative sequelae in families of autistic children, limited attention has been devoted to stress reduction interventions for this population. The comparative lack of research may stem in part from initial assumptions that interventions designed to support child functioning and reduce co-occurring behavior problems would inherently alleviate parenting stress. Unfortunately, parenting stress often remains unchanged in the context of such interventions (see Oono et al., 2013 for a review). Growing recognition of the importance of intervening directly with parenting stress has led to a recent increase in the development and testing of targeted stress-reduction intervention.

Historically, stress-reduction efforts have commonly employed parent support groups (Hastings & Beck, 2004). Community-based implementation of psychoeducational support is widespread, and preliminary research suggests potential benefits for reducing stress and improving mental health in parents of children with developmental disabilities (Bourke-Taylor et al., 2021; Hastings & Beck, 2004). However, efficacy data are limited by frequent reliance on non-experimental or quasi-experimental designs, and the use of heterogeneous samples that preclude population-specific inferences (Bourke-Taylor et al., 2021; Hastings & Beck, 2004; Rutherford et al., 2019). Stringent, randomized trials with well-characterized and representative samples are needed in order to clarify the utility of psychoeducational support groups for targeted stress reduction in parents of young autistic children.

Other recent stress-reduction approaches have embraced a mindfulness framework, emphasizing management rather than elimination of chronic stress. Mindfulness-based hybrids, such as Mindfulness-Based Cognitive Therapy (MBCT; Segal et al., 2002) and Mindful Parenting (Bögels & Restifo, 2014), have demonstrated efficacy for reducing parenting stress (Ferraioli & Harris, 2013; Jones et al., 2018), enhancing stress reappraisal (Rayan & Ahmad, 2016), and improving mental health in parents of autistic children (Ridderinkhof et al., 2018; Schwartzman et al., 2022). Fewer studies have examined traditional Mindfulness-Based Stress Reduction (MBSR; Kabat-Zinn, 2013) in this population, even though MBSR is the most empirically supported stress-reduction intervention to date (e.g., de Vibe et al., 2017; Kriakous et al., 2021; Zhou et al., 2020). Some investigations of MBSR have used mixed samples comprising parents of autistic children and parents of children with other heterogeneous IDD diagnoses (Dykens et al., 2014; Neece, 2014), while others have tested MBSR in combination with additional interventions (Rojas-Torres et al., 2021; Weitlauf et al., 2020). Results regarding the efficacy of MBSR for parents of children with neurodevelopmental disorders are promising, but further investigation is necessary to demonstrate the efficacy and unique effects of MBSR for parents of autistic children specifically. Furthermore, existing MBSR research has minimally attended to racial, ethnic, and socioeconomic diversity, leading to calls for greater inclusivity, especially for Latinx and low-income communities (Lenger et al., 2022; Nagy et al., 2022). With some exceptions (e.g., Neece et al., 2019), studies of MBSR in families of autistic children have predominantly included White, Non-Hispanic participants. Extensions to diverse samples are overdue and necessary to enhance external validity, generalizability, and eventual dissemination.

Efforts to promote service equity have increasingly turned to telehealth as an avenue for improving access and utilization in underserved families of autistic children (Stuckey & Domingues-Montanari, 2017). Virtual interventions demonstrate good feasibility, high acceptability, and generally comparable efficacy to in-person interventions targeting a range of needs in autistic children and their families (for reviews see Boisvert et al., 2010; Ellison et al., 2021; Sutherland et al., 2018). Only a few studies have piloted virtual delivery of mindfulness-based hybrids for parents of autistic individuals (Kuhlthau et al., 2020; Lunsky et al., 2021), with some evidence of similar outcomes across MBCT modalities for parents of adolescents and adults (Lunsky et al., 2021). To our knowledge, no study has examined virtual delivery of MBSR for families of autistic individuals.

The Stress-reduction Techniques for Enhancing Parenting Skills (STEPS) randomized controlled trial tested the efficacy of MBSR relative to active Psychoeducational Support (PE) for diverse and underserved parents of autistic preschoolers. In a prior publication (Neece et al., 2023), we demonstrated that MBSR was superior to PE in reducing parenting stress, with group differences significant at 1-year follow-up. Strengths of the STEPS trial include a large and well-characterized sample, involvement of children with a range of developmental and behavioral needs, and long-term follow-up at 6 and 12 months post-intervention.

In this paper, we detail implementation indicators (Orsmond & Cohn, 2015; Proctor et al., 2011) to provide further insight into the intervention experiences of diverse families historically underrepresented in autism and mindfulness research. Additionally, although we initiated an in-person investigation, the onset of the COVID-19 pandemic necessitated a mid-trial pivot to virtual intervention, providing an unexpected opportunity to consider intervention modality in relation to implementation indicators and intervention efficacy as well. Drawing upon existing evidence (Boisvert et al., 2010; Ellison et al., 2021; Kuhlthau et al., 2020; Lunsky et al., 2021; Sutherland et al., 2018), we expected that virtual delivery might ease access and thereby improve attendance, but we did not expect modality to significantly affect intervention outcome.

## Method

### Participants

Primary outcomes and methods from the STEPS randomized controlled trial are detailed in Neece et al. (2023). Participants included 117 families of autistic children aged 3 to 5 years recruited from the community from September 2018 to March 2021. Three cohorts participated, with assessments at baseline, immediately post-intervention (8 weeks), and at 6- and 12-months post-intervention. Initially designed as an in-person study, the project pivoted to a virtual modality following the onset of the COVID-19 pandemic. Virtual follow-up assessments were necessitated for cohort 2 at 6 and 12 months. Cohort 3 participated in a fully virtual experience, with the exception of direct assessments of child IQ and receptive language, which were completed once in-person activities resumed post-intervention. Parents participating virtually were provided with any necessary technology, including devices and internet access.

Trial inclusion criteria involved (a) child community diagnosis of autism spectrum disorder (ASD)—or waitlisted for a community ASD assessment—with diagnostic symptoms verified by study-administered assessments, (b) child age 3 to 5 years, and (c) parent ability to complete study procedures in English. Exclusionary criteria were as follows: (a) positive screen for active parental psychosis, substance abuse, or suicidality according to the associated modules of the Structured Clinical Interview for DSM Disorders (First et al., 2002); (b) parent participation in an auxiliary mental health treatment or support group at time of randomization; and (c) child motor impairment that would prevent participation in the broader assessment protocol (e.g., difficulty sitting independently).

The majority of participating primary caregivers were mothers (91%) who identified as Latinx (51%) and were married or living with a partner (76%). Primary caregivers were 34.6 years of age on average (*SD* = 7.5). About one-quarter of primary caregivers (24%) reported an education level of high school or less, and almost half of the families (44%) had an annual household income below US $50,000. At baseline, primary caregivers reported significant distress, with 52% reporting elevated levels of parenting stress (≥ 85^th^ percentile; *M* = 37.5, *SD* = 8.7) on the Parental Distress subscale of the Parenting Stress Inventory-4-Short Form (PSI4-SF; Abidin et al., 2006), and 51% endorsing clinically elevated depressive symptoms on the Center for Epidemiological Studies – Depression Scale (≥ 16; CES-D; Radloff, 1977). Most participating children were boys (80%), with an average age of 52.5 months (*SD* = 11.0). The majority of the children (68%) met DSM-5 criteria for co-occurring ID (IQ and adaptive behavior < 76; American Psychiatric Association, 2013), and presented with clinically elevated parent-reported total behavior problems on the Child Behavior Checklist (81%; CBCL; Achenbach, 2009). Table 1 presents detailed participant demographic and clinical characteristics by intervention modality.

### Procedure

Interested parents contacted the project team by phone, postcard, or the study website. Following an initial phone screening, eligible families were scheduled for a baseline assessment and parents provided informed consent.

At the baseline visit, parents were interviewed about family demographics, service utilization, and child adaptive behavior (Vineland Adaptive Behavior Scales-3; Sparrow et al., 2016). Parents also completed a battery of questionnaires and participated in parent–child interactive tasks (not used in the current study). Direct assessments of child cognitive functioning (Stanford-Binet Intelligence Scales-5 ABIQ; Roid, 2003), receptive language (Peabody Picture Vocabulary Test-4; Dunn & Dunn, 2007), and ASD symptoms were performed. For Cohorts 1 and 2, ASD diagnostic confirmation was completed through a multi-method assessment involving administration of a standardized parent-report form, the Social Communication Questionnaire (SCQ; Rutter et al., 2003a), and direct testing with the Autism Diagnostic Observation Schedule-2 (ADOS-2; Lord et al., 2012). Procedures for cohort 3 were modified due to pandemic-related restrictions on in-person activities. For Cohort 3, ASD diagnostic status was ascertained using two standardized parent-report questionnaires—the SCQ (Rutter et al., 2003a) and the Social Responsiveness Scale-2 (SRS-2; Constantino & Gruber, 2012)—and a comprehensive semi-structured parent interview, the Autism Diagnostic Interview-Revised (ADI-R; Rutter et al., 2003b). Additionally, assessments of child cognitive functioning and receptive language were conducted post-intervention for Cohort 3, once in-person activities resumed. Neece et al. (2023) contains additional details regarding diagnostic confirmation and clinical best estimate procedures.

**Fig. 1:**
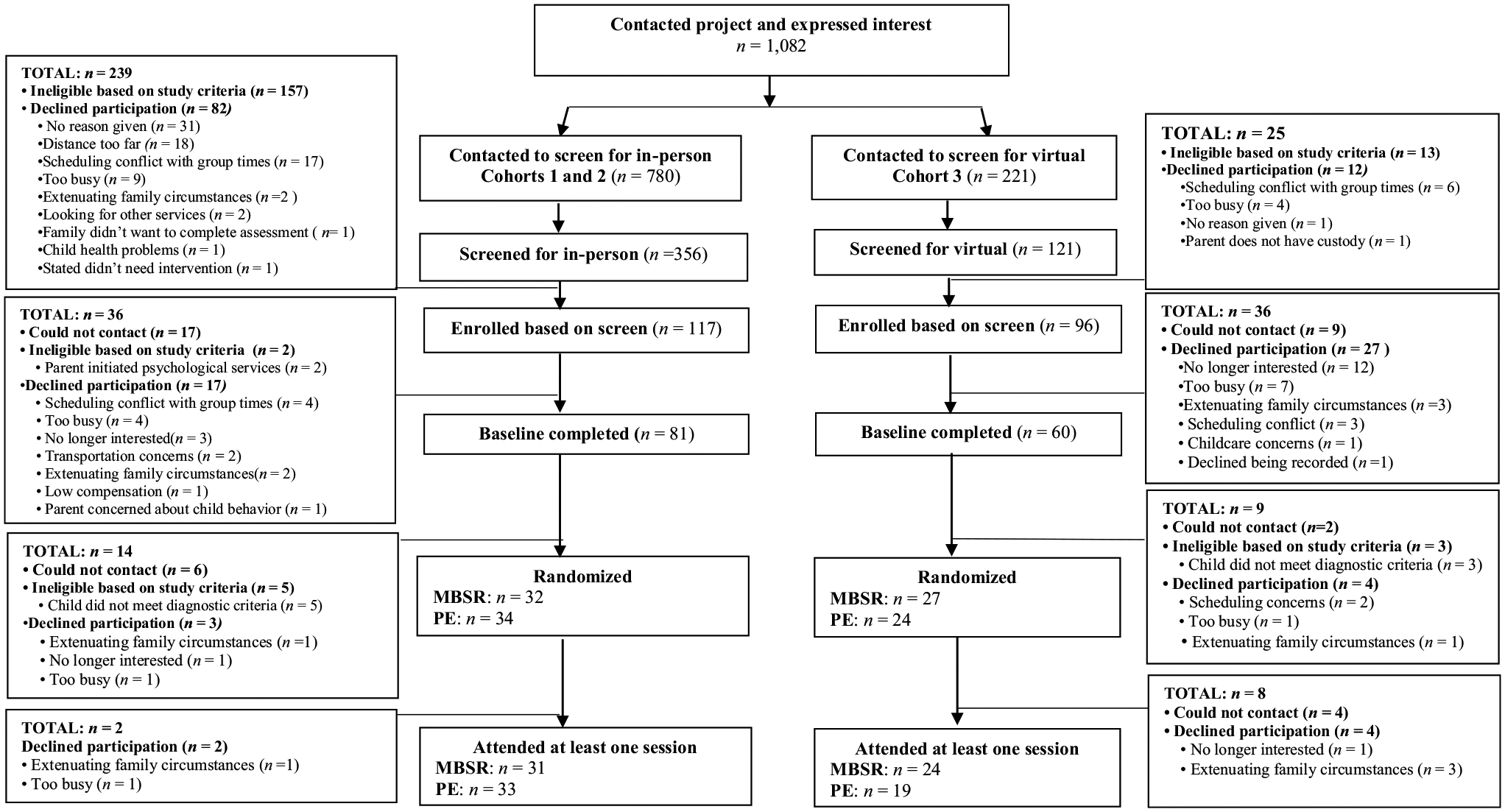
CONSORT diagram

Following completion of the baseline assessment, families were randomly assigned to either MBSR (*n* = 59) or PE (*n* = 58). Figure 1 presents the CONSORT diagram and the flow of participants through the study according to in-person (MBSR: *n* = 32; PE: *n* = 34) and virtual cohorts (MBSR: *n* = 27; PE: *n* = 24).

#### Participation Enhancement

At the conclusion of the baseline visit, we implemented an adapted version of Nock and Kazdin’s (2005) *Participation Enhancement Intervention* (PEI) based on similar procedures utilized by Fenning and colleagues (Fenning et al., 2022a, b). Our adapted PEI is a brief motivational interviewing module designed to optimize intervention engagement and reduce barriers anticipated to be heightened for our underserved families. We worked individually with parents for 10 to 30 min to develop a collaborative plan to promote parent-identified intervention goals and to proactively address potential intervention barriers (e.g., barriers to attendance, persistence, utilization, and home practice). PEI sessions were delivered as needed to support engagement throughout intervention, with planned boosters conducted prior to Session 6 and at the conclusion of the intervention to promote involvement in longitudinal follow-up.

#### MBSR Intervention

Following the established manual (Kabat-Zinn, 2013), MBSR included eight weekly 2-hr group sessions, a day-long 6-hr meditation retreat on the weekend after Session 6, 30–45 min of daily home practice guided by instructional audio, and a parent workbook. Formal mindfulness exercises aimed to increase present-moment awareness with a compassionate, non-judgmental stance, and included body scans, mindful yoga, and sitting meditation. Participants were also taught to practice mindfulness informally in everyday activities. During groups, participants practiced formal mindfulness exercises, broke into dyads to discuss daily homework practice, and met as a larger group to discuss topics related to the practice of mindfulness in everyday life. The MBSR intervention sessions and retreat were delivered by a certified MBSR instructor with over 20 years of experience and co-led by clinical psychology doctoral students who had experience with MBSR and received weekly supervision with the certified instructor.

#### Psychoeducational Support (PE)

The active PE comparator ran concurrently with the MBSR group, and was conducted at the same time and location, but on different days to avoid contamination. The PE condition consisted of eight weekly 2-hr sessions; a day-long 6-hr family resource fair after Session 6; daily homework that included monitoring progress on goals identified at the end of each session; and a workbook for parents that provided information regarding their child’s development, diagnosis, and associated considerations. To enhance external validity, the PE group was modeled after support groups offered to parents of autistic children in the local community. Each session had a general topic for discussion such as preparing for individualized education plan meetings, parental advocacy, sibling issues, social support, and transition to kindergarten. At the start of each session, group leaders provided didactic instruction on the topic, then facilitated small- and large-group discussions. PE group sessions were led by parents with lived experience who were identified as local community leaders working in the field. Clinical psychology doctoral students co-led PE groups and received weekly supervision from the parent group leader and a licensed clinical psychologist.

#### Virtual Intervention Modifications

Virtual interventions were delivered using the Zoom videoconferencing platform. Participants were provided with group-specific links and passwords to help ensure confidentiality in the virtual setting. Additionally, project staff monitored Zoom sessions to admit and manage participants, and to provide technological support as needed. Group leaders adhered to the respective MBSR and PE manuals when delivering intervention content, though some aspects of group process were modified for the virtual setting. For example, during the first session, group leaders briefly introduced the Zoom platform and relevant features, and reviewed expectations for online interaction. Zoom break-out rooms were employed to facilitate dyadic and small-group discussions for both MBSR and PE virtual groups, and the whiteboard was used as a visual support when needed. The 6-hr meditation retreat (MBSR) and the resource fair (PE) were also conducted via Zoom. Barriers to participation in virtual interventions were addressed using our adapted PEI, in a manner consistent with our in-person interventions.

#### Childcare

Childcare was provided during all in-person MBSR and PE sessions. Undergraduate and graduate student childcare providers were trained to provide adequate support for autistic children and their siblings without delivering behavioral intervention. Childcare providers received weekly supervision from a licensed clinical psychologist. Childcare was not provided during virtual intervention for Cohort 3 due to pandemic-related prohibitions on in-person activities, but we did troubleshoot any related barriers to participation using our adapted PEI.

#### Follow-up Assessments

Follow-up assessments occurred immediately post-intervention and at 6 and 12 months post-intervention. Demographics and service information were updated, and parents again completed the questionnaire packets.

### Measures

#### Demographic Information

Parents reported on child and parent age, race, ethnicity, family income, child diagnoses, and services accessed/utilized via interview.

#### Parenting Stress

To index the construct of parenting stress comprehensively, we assessed three forms of parenting stress: general distress, stress specific to the autistic child, and daily parenting hassles. The Parental Distress subscale of the Parenting Stress Index-4, Short Form (PSI4-SF; Abidin et al., 2006) assessed perceived general distress in the parenting role (study Cronbach’s *α*-values = 0.83–0.88). The Negative Impact scale of the Family Impact Questionnaire (FIQ; Donenberg & Baker, 1993) assessed stress specific to the autistic child relative to the impact of other same-aged children (study Cronbach’s *α*-values = 0.87–0.92). The Intensity subscale of the Parenting Daily Hassles (PDH; Crnic & Greenberg, 1990) assessed perceived intensity of daily stressors related to caregiving demands and responsibilities (study Cronbach’s *α*-values = 0.90–0.94). For all three measures of parenting stress, higher scores indicate greater endorsement of parenting stress.

#### Intervention Fidelity

Throughout delivery of the intervention, trained observers monitored MBSR and PE groups for adherence to intervention targets. Intervention fidelity scores were calculated according to the percentage of intervention components completed as outlined in the respective MBSR and PE manuals. Total contact minutes were also recorded.

#### Intervention Attendance

Weekly attendance data were recorded for all participating primary caregivers. We considered attendance continuously, as indexed by the total number of sessions attended, and dichotomously according to the proportion attending at least one session and the proportion attending the majority of sessions (≥ 4 of 9 weeks). We also recorded whether a second caregiver elected to attend group sessions alongside the primary caregiver.

#### Intervention Adherence

At each intervention session, parents reported on the degree of past-week homework completion using a 3-point scale: 0 = *not completed*, 1 = *partially completed*, or 2 = *completed*. For purposes of analyses, we collapsed across the latter two categories. We considered adherence both continuously (total number of weeks of attempted/completed homework) and dichotomously (majority of assignments attempted/completed; ≥ 4 of 9 weeks).

#### Intervention Satisfaction

At the final intervention session, parents reported on their overall satisfaction with the intervention program, the ease or difficulty of the intervention program, and the usefulness of the intervention program using an adapted version of the Parent Satisfaction Questionnaire (McIntyre, 2008). Items were rated on a 7-point Likert scale, with higher numbers indicating greater satisfaction, ease, or utility. Satisfaction data are summarized as the proportion designating the highest two levels of satisfaction (i.e., improved/very improved, easy/extremely easy, useful/extremely useful).

#### Telehealth Satisfaction

For Cohort 3 only, parents also reported on their overall satisfaction with the telehealth experience at the final intervention session. Telehealth satisfaction items were rated on a 7-point Likert scale, with higher numbers indicating greater satisfaction. Satisfaction data are summarized as the proportion designating the highest two levels of satisfaction (e.g., positive/very positive, easy/extremely easy). Parents also reported on preferences and challenges related to the telehealth modality using a dichotomous yes/no format, and indicated likelihood of accessing future services online (yes/unsure/no).

### Data Analyses

Descriptive data for session attendance, adherence, and intervention satisfaction were examined. Comparisons for the full sample were performed across intervention conditions (MBSR vs. PE). Modality comparisons (in-person vs. virtual) were performed within intervention conditions. Categorical variables were compared between groups using chi-square tests and continuous variables were compared using *t*-tests. Quantitative satisfaction data are summarized as the proportion designating the highest two levels of satisfaction.

The role of intervention modality on rate of change in parenting stress was examined using a two-level linear growth curve model. Analyses were intent-to-treat. Repeated measures across time at Level 1 were nested within individuals (primary caregivers) at Level 2. Time was defined according to study time points (Baseline, Post-Intervention, 6-month Follow-up, 12-month Follow-up), and was centered at baseline to improve interpretability. The FIQ-NI was used to set the metric for the parenting stress latent variable, which was defined by three indicator variables: the PSI-PD, FIQ-NI, and PDH. The data met the assumptions of multilevel modeling (e.g., Singer & Willett, 2003). Detailed missing data analyses as well as estimation and imputation methods using Blimp 3 (Keller & Enders, 2021) are reported in Neece et al. (2023). In brief, there were no significant differences in attrition between intervention groups, intervention modalities, or cohorts. However, preliminary analyses revealed consistent associations over time between outcome variables and the following Level 2 variables, which were entered as covariates: family utilization of mental health services in the 6 months prior to baseline (0 = *No*, 1 = *Yes*) and number of months of applied behavior analysis for the target child in the previous 6 months. Covariates were centered to improve interpretability.

As reported in Neece et al. (2023), a model in which intervention group predicted change in parenting stress over time fit the data better than the unconditional means model, the unconditional growth model, a model in which intervention group predicted baseline parenting stress, and a model in which Level 2 covariates predicted baseline parenting stress in addition to intervention group. To evaluate the effect of intervention modality, we further expanded upon the best-fitting model from Neece et al. (2023) (Model 1), by testing a model with a two-way interaction between intervention modality and change in parenting stress over time (Model 2), and a model with a three-way interaction between intervention type, intervention modality, and change in parenting stress over time (Model 3). The latter two models were tested to determine whether each model fit the data significantly better than the previous model.

## Results

### Participant Characteristics

MBSR and PE groups did not differ on the variables presented in Table 1 (see Neece et al., 2023). However, examination across modalities within intervention conditions revealed differences in parenting stress for the MBSR in-person and virtual groups. Specifically, parents receiving in-person MBSR reported higher levels of stress relative to parents receiving virtual MBSR according to both the PSI-PD, *t*(51) = 2.08, *p* = 0.043, and the FIQ-NI, *t*(51) = 2.07, *p* = 0.043. However, neither stress scale was associated with indicators of MBSR implementation, so no further control was required in relevant analyses.

### Intervention Fidelity by Modality

Fidelity was similar and did not differ significantly between in-person MBSR (97% of intervention content items delivered) and virtual MBSR (100%), *t*(1) = −2.02, *p* = 0.293. Total contact time for MBSR groups also did not differ significantly by modality, with in-person MBSR averaging 20.2 hr (*SD* = 0.37), and virtual MBSR averaging 21.9 hr, *t*(1) = −3.63, *p* = 0.171. Fidelity and contact time were comparable across in-person and virtual PE as well. Both in-person and virtual PE completed 98% of possible intervention delivery items, *t*(1) = −0.58, *p* = 0.666. Average total contact time did not differ by PE modality (in-person PE = 17.3 hr, *SD* = 2.57 vs. virtual PE = 20.8 hr, *t*[1] = −1.09, *p* = 0.472).

### Intervention Attendance and Adherence

Considering the sample as a whole, nearly all participants attended at least one session (95% MBSR vs. 90% PE, *χ*^2^[1] = 1.14, *p* = 0.286), and most attended the majority of sessions (≥ 4 of 9 sessions; 79% MBSR vs. 88% PE, *χ*^2^[1] = 1.90, *p* = 0.168). MBSR participants averaged 6.3 total sessions (*SD* = 2.7) and PE participants averaged 7.0 total sessions (*SD* = 2.2), *t*(104) = 1.50, *p* = 0.068. A quarter of the MBSR families and 36% of the PE families had a second caregiver attend sessions, *χ*^2^(1) = 1.60, *p* = 0.206. Families were relatively engaged with respect to homework, with most parents attempting or completing homework for at least 4 of the 9 weeks (75% MBSR vs. 85% PE, *χ*^2^[1] = 1.54, *p* = 0.215). Over the course of the intervention, MBSR parents averaged 4.9 weeks (*SD* = 2.2) of homework completion and PE parents averaged 5.4 weeks (*SD* = 2.0) of homework completion, *t*(96) = 1.06, *p* = 0.292. There were no significant differences in attendance or adherence between the MBSR and PE groups, suggesting similar levels of engagement across intervention conditions.

Table 2 presents comparisons of attendance and adherence data across virtual and in-person modalities within intervention conditions. On the whole, a greater proportion of parents attended at least one session when the interventions occurred in person as opposed to online; this difference was significant for PE. Secondary caregivers were also significantly more likely to attend in-person groups. However, parents were significantly more likely to persist in virtual intervention according to multiple indicators of attendance for both MBSR and PE. Rates of homework completion did not differ significantly by modality.

### Modality and Intervention Efficacy

**Fig. 2:**
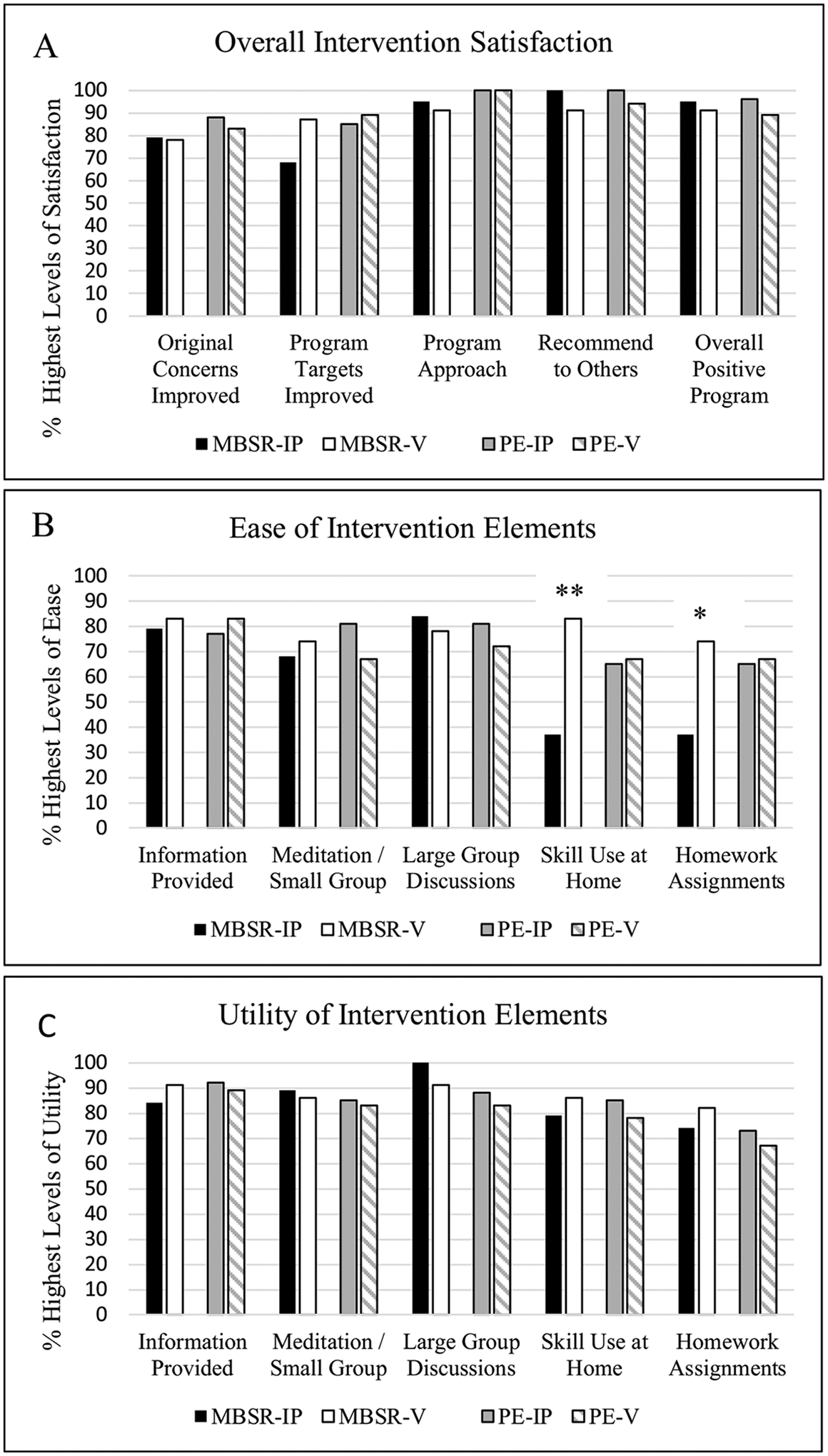
Reported intervention satisfaction, ease, and utility

Table 3 presents the results of multi-level model testing. As reported previously in Neece et al. (2023) and as reflected in Table 3, parenting stress decreased significantly for both groups, but MBSR resulted in significantly greater stress reduction than did PE. Subsequent models testing the interaction between intervention modality and change in parenting stress over time (Model 2), as well as a three-way interaction between intervention type, modality, and change in parenting stress over time (Model 3), were non-significant, *p*-values > 0.05. Therefore, change in parenting stress did not depend on modality, nor did it depend on the interaction between intervention type and modality (Fig. 2).

### Satisfaction and Utilization

The sample as a whole reported relatively high levels of program satisfaction. Ninety-five percent of MBSR participants and 98% of PE participants indicated that they would recommend or strongly recommend the program to others, *χ*^2^(1) = 0.395, *p* = 0.529. Ninety-three percent of participants in each group reported positive or very positive feelings about the program, *χ*^2^(1) = 0.003, *p* = 0.953. Additionally, 79% of the MBSR group and 86% of the PE group reported improvement in the concerns that originally led them to participate in the STEPS program, *χ*^2^(1) = 0.906, *p* = 0.341.

Parents also reported on the difficulty of MBSR and PE program elements. A majority of participants endorsed the following as easy or very easy: information presented in group (81% MBSR, 80% PE, *χ*^2^[1] = 0.027, *p* = 0.870), MBSR-guided meditations/PE small-group discussions (71% MBSR, 75% PE, *χ*^2^[1] = 0.140, *p* = 0.708), large-group discussions (81% MBSR, 77% PE, *χ*^2^[1] = 0.176, *p* = 0.675), skills at home (62% MBSR, 66% PE, *χ*^2^[1] = 0.149, *p* = 0.699), and homework (57% MBSR, 66%, PE, *χ*^2^[1] = 0.698, *p* = 0.403). Parents rated the utility of program components as well. Again, most identified program components as useful or extremely useful: information presented in group (90% MBSR, 91% PE, *χ*^2^[1] = 0.005, *p* = 0.945), MBSR-guided meditations/PE small-group discussions (88% MBSR, 84% PE, *χ*^2^[1] = 0.241, *p* = 0.623), large-group discussions (95% MBSR, 86% PE, *χ*^2^[1] = 1.909, *p* = 0.167), skills at home (83% MBSR, 82% PE, *χ*^2^[1] = 0.018, *p* = 0.893), and homework (78% MBSR, 70% PE, *χ*^2^[1] = 0.638, *p* = 0.424). Taken together, results suggest strong overall acceptability and utility of MBSR and PE for these diverse families.

**Fig. 3:**
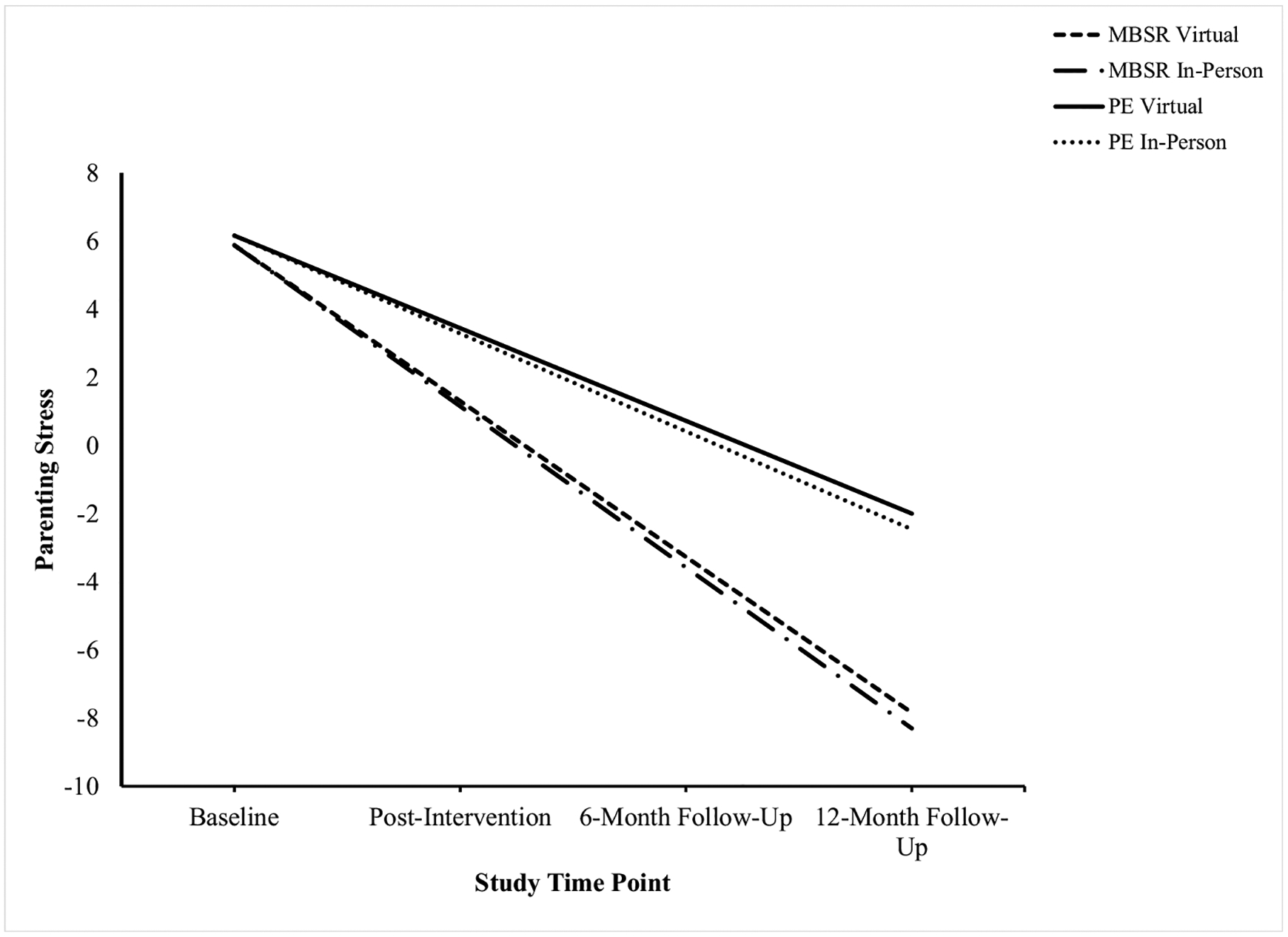
Change in parenting stress by intervention type and modality. MBSR, Mindfulness-Based Stress Reduction. PE, psychoeducational support. Graph is adjusted for family mental health services in the 6 months prior to baseline and number of months of applied behavior analysis (ABA) at baseline. Parenting stress latent variable scores are reported

Indicators of general program satisfaction did not differ significantly by modality, with parents in MBSR and PE reporting generally high levels of satisfaction overall regardless of in-person or virtual format, *p*-values > 0.05 (Fig. 3). However, significant differences in particular aspects of program difficulty were observed for MBSR families. As displayed in Fig. 2 (Panel B), participants in virtual MBSR reported significantly greater ease in using skills at home (virtual MBSR = 83% vs. in-person MBSR = 37%, *χ*^2^[1] = 9.24, *p* = 0.002) and in completing homework assignments than did parents participating in person (virtual MBSR = 74% vs. in-person MBSR = 37%, *χ*^2^[1] = 5.84, *p* = 0.016). No other differences emerged in program difficulty or utility across modalities for either MBSR or PE, suggesting relatively high, consistent acceptability and utilization, *p*-values > 0.05.

Of the 51 families who participated in virtual MBSR or virtual PE, 38 returned a completed telehealth satisfaction survey. Parents largely reported positive to very positive experiences with the online programs (85% MBSR, 83% PE), and almost universal benefit of online participation (95% MBSR, 100% PE). Parents endorsed a number of specific benefits of virtual participation, including the ability to remain at home (74% MBSR, 83% PE), not having to drive to groups (68% MBSR, 83% PE), the opportunity to interact with other parents online (79% MBSR, 78% PE), the nature of the group leader’s engagement online (58% MBSR, 61% PE), the nature of online group discussions (68% MBSR, 61% PE), the option to turn off or mute video as needed (89% MBSR, 78% PE), and increased ease of partner participation in online groups (11% MBSR, 28% PE). A comparatively small number of parents (*n* = 15) reported challenges with online participation, including the lack of in-person interaction (43% MBSR, 43% PE), difficulties with childcare (43% MBSR, 43% PE), trouble juggling demands (57% MBSR, 71% PE), and limited privacy (43% MBSR, 29% PE). Although the project ensured that all parents had access to a computer or smart device and internet for groups, some (32% MBSR, 11% PE) nonetheless experienced technical or equipment difficulties such as slow or disrupted internet, problems logging in, and challenges with the Zoom platform. About half of the participating parents indicated a preference for virtual services (45% MBSR, 56% PE), with a majority indicating a willingness to pursue virtual services in the future if needed (70% MBSR, 72% PE). Most indicated that the virtual experience with the STEPS program increased their comfort with online clinical services (64% MBSR, 73% PE).

## Discussion

Utilizing a rigorous design involving an active comparator, multiple measures of parenting stress, and long-term follow-up, the STEPS randomized controlled trial demonstrated the efficacy of MBSR (Kabat-Zinn, 2013) for reducing parenting stress in families of young autistic children during critical years for early intervention (Neece et al., 2023). MBSR outperformed PE, though PE also conferred benefits, highlighting the value of high-quality information and social support, and lending credence to existing community programs that served as a model for our PE program. In the current paper, we leveraged an unexpected pandemic-mandated pivot to virtual intervention to evaluate the effects of modality on intervention efficacy. Consistent with a growing body of work suggesting comparable efficacy of virtual and in-person interventions for families of autistic children (Boisvert et al., 2010; Ellison et al., 2021; Sutherland et al., 2018), modality did not significantly affect MBSR or PE outcomes. Preliminary evidence of similar efficacy across virtual and in-person formats suggests the potential to expand access to much needed stress-reduction interventions through use of remote technologies.

In line with calls to address historic underrepresentation of marginalized populations in autism intervention science (Harris et al., 2020; Steinbrenner et al., 2022; West et al., 2016), we emphasized the inclusion of racially, ethnically, and socioeconomically diverse participants in our randomized controlled trial. In addition, a high proportion of the children in our sample presented with co-occurring intellectual disability, a subpopulation that is also significantly underrepresented in autism research (Russell et al., 2019). Optimizing intervention efficacy and implementation for diverse autistic children and their families is essential to reducing access barriers and to remediating intervention disparities.

To enhance understanding of processes central to engaging and serving diverse families, we examined implementation indicators. Over 1000 families contacted our project to express interest in participating in our program. We found partnering with community-based agencies and service providers to be a particularly effective outreach strategy; we also benefitted from word-of-mouth and self-referrals. Although we dedicated significant effort to recruitment endeavors, our relative ease in achieving enrollment targets underscores the high level of community need for parent stress-reduction supports. Feasibility of our interventions is further supported by strong attendance and retention data. Nearly all participants attended at least one session of MBSR or PE, and most attended the majority of group sessions. To help remediate heightened barriers for our underrepresented and underserved families, we proactively threaded engagement interventions throughout treatment and follow-up using an adapted version of Nock and Kazdin’s (2005) PEI (Fenning et al., 2022a, b). We found our adapted PEI to be an effective mechanism for individualizing supports and troubleshooting barriers in the context of group intervention.

Notably, persistence was better for virtual groups than it was for in-person groups. Although we provided families with transportation assistance for in-person sessions as needed, online delivery of intervention may have further remediated barriers to attendance for primary caregivers. Conversely, the reverse attendance pattern was observed for optional attendance of second caregivers, who were more likely to join in person. We suspect that child care needs may account for this difference, as project staff provided child care during in-person groups, but child care remained the province of families during virtual groups. Researchers and practitioners should weigh evidence of modality and location effects on attendance patterns when designing future programs and rendering referrals. When possible, providing flexibility and choice in modality and location may optimize intervention participation, especially for underserved populations. Indeed, a previous randomized controlled trial involving families of autistic children with similar demographics found that providing families with flexibility in the location of intervention sessions, including the ability to shift sessions from clinic to home, appeared central to promoting parent engagement (Fenning et al., 2022a, b).

Parents in MBSR and PE endorsed high levels of overall program satisfaction, which did not differ by intervention type or modality. Anecdotally, concerns regarding use of MBSR for parents of autistic children have often centered upon the length of the intervention sessions, feasibility of homework requirements, and expectations for daily meditation practice. We carefully matched MBSR and PE for homework time and intensity, and parents in both groups attempted or completed homework most weeks. There were no significant differences between MBSR and PE in reported homework adherence, ease, or utility. Although parents generally endorsed homework as easy and useful, it was the least preferred aspect of our program for both groups. However, important modality effects emerged for MBSR. Specifically, parents participating in virtual MBSR reported significantly less difficulty completing homework than parents attending MBSR in person. Virtual MBSR participants also reported greater ease in using MBSR skills at home than did parents attending in person. It is possible that virtual delivery of MBSR may enhance contextual learning in the home environment, increasing familiarity and comfort with home practice and creating a natural bridge to homework completion and integration of MBSR techniques in everyday life. Subsequent analysis of parents’ qualitative comments may provide further insight into parent experiences, and it will be important to understand whether virtual MBSR might confer particular benefits for rehearsal and utilization of learned skills.

The COVID-19 pandemic has accelerated the embrace of telehealth and telepsychology. The families participating in our virtual interventions generally reported positive experiences with online participation. Parents identified several benefits unique to the virtual experience, such as the ability to remain at home and not drive to groups, and the ability to turn off or mute video. Challenges with virtual participation were also noted, including difficulty with childcare, trouble juggling demands, and limited privacy. Additionally, some parents reported missing in-person interaction, though this was not unique to our program given concurrent COVID-19 mandates against in-person activities. On the whole, most parents reported a willingness to pursue virtual services in the future, with about half indicating a preference for virtual services. These data suggest potential avenues for further refining virtual programs to fit the needs of underserved families.

### Limitations and Future Research

This study has many strengths, including a rigorous randomized design with an active comparator, a well-characterized and diverse sample, multi-measure assessment of stress, and long-term follow-up. However, important limitations remain. First and foremost, the onset of the COVID-19 pandemic occurred mid-trial. Although we did not detect cohort differences, this world-changing event undoubtedly affected our participating families and altered the context for this stress-reduction study. In addition, our sample is relatively large for randomized trials involving families of autistic children, but subsamples for virtual groups were comparatively small and power may have been limited. It will be important to replicate effects in a study powered specifically to test effects of intervention modality. Furthermore, we designed this study as a stringent test of MBSR using an active PE comparator. We did not employ a no-intervention or waitlist control given concerns regarding risks of untreated stress (Schwartzman et al., 2022), especially for high-risk, underserved families. However, this design prevents evaluation of the relative merits of MBSR and PE compared to the natural unfolding of parenting stress over time.

Lastly, although results revealed strong implementation of traditional MBSR for our diverse families, MBSR is an intensive, multi-component intervention. Future dismantling efforts might assist in refining intervention to include the most essential elements. We attempted to individualize the group experience through use of a motivational interviewing module (Fenning et al., 2022a, b; Nock & Kazdin, 2005). Considering additional avenues for further tailoring MBSR by attending to intersectionality and the cultural (e.g., Nagy et al., 2022), socioeconomic (e.g., Lenger et al., 2022), and population-specific adaptations that may benefit diverse families of young autistic children will be important. Moreover, though research is needed to clarify the potentially distinct mechanisms and advantages of MBSR and PE, it is possible that combining these approaches may be especially helpful to parents of young autistic children who are newly navigating a complex parenting role. Testing the possible additive effect of these stress-reduction interventions may be especially helpful for families from underserved and marginalized backgrounds where resource and clinical needs are greater.

MBSR and PE appear feasible, acceptable, and efficacious for diverse and underserved parents of young autistic children. Preliminary evidence of comparable efficacy across virtual and in-person modalities suggests the potential to further expand access to vital stress-reduction interventions through use of telehealth technology.

## Figures and Tables

**Table 1:** Demographic and clinical comparisons across modalities within intervention condition

Variable	MBSR		PE	
	In-Person (*n* = 32)	Virtual (*n* = 27)	In-Person (*n* = 34)	Virtual (*n* = 24)
*Child characteristics*				
Male (%)	78	78	79	88
Mean age in months (*SD*)	52.1 (10.8)	52.9 (10.4)	53.5 (13.4)	51.1 (8.5)
Race/ethnicity (%)				
White	25	19	18	17
Latinx	44	37	41	58
Black	0 +	11 +	9	0
Asian	3	4	9	8
Pacific Islander	0	0	0	4
Other	3	11	0	4
Multi-racial	25	19	24	8
Mean IQ (*SD*)	65.0 (16.8) +	80.2 (30.9) +	65.1 (18.1)	73.3 (21.0)
Intellectual Disability Status (%)	69	70	71	58
Mean Adaptive Behavior (*SD*)	67.5 (10.8)	69.1 (9.4)	68.9 (8.7)	70.3 (9.3)
Mean SCQ total score (*SD*)	21.0 (4.4)	22.8 (6.4)	20.3 (5.3)	20.4 (6.1)
CBCL Total Problems *T*-Score (*SD*)	72.7 (9.7)	72.2 (12.0)	69.8 (9.6)	69.3 (10.2)
Clinically Elevated (*T* > 63; %)	84	86	79	74
*Primary caregiver characteristics*				
Female (%)	91	96	85	92
Mean age in years (*SD*)	33.7 (5.3)	35.6 (9.5)	35.2 (8.3)	33.9 (6.4)
Race/ethnicity (%)				
White	22	19	24	21
Latinx	59	48	44	54
Black	0 +	11 +	12 +	0 +
Asian	3	4	9	8
Pacific Islander	0	0	0	4
Other	0	7	0	4
Multi-racial	16	11	12	8
Education level (%)				
High school or less	28	35	18	17
Some college	16	31	21	21
Technical degree/AA	28	8	41	25
Bachelor’s degree	16	12	6	29
Graduate degree	13	15	15	8
Marital status (%)				
Married	56	56	65	71
Living together	19	11	18	8
Separated	3	4	9	8
Divorced	0	4	0	0
Widowed	0	4	3	4
Single	22	22	6	8
Mean Depression Score (*SD*)	22.2 (12.4) +	15.7 (12.4) +	17.6 (9.2)	15.7 (10.7)
Parenting stress				
Mean Parental Distress (PSI-SF) (*SD*)	39.6 (9.0)*	34.5 (8.7)*	38.6 (7.6)	35.3 (9.4)
Mean Negative Impact (FIQ) (*SD*)	40.4 (12.0)*	32.6 (15.5)*	37.2 (13.3)	34.9 (11.5)
Mean Hassles Intensity (PDH) (*SD*)	62.7 (16.9)	55.4 (17.6)	55.6 (18.2)	62.8 (21.2)
*Family-level characteristics*				
Annual gross family income (%)				
< US $30 K	31	19	30	25
US $30,000 to < US $50,000	16	22	12	20
US $50,000 to < US $70,000	19	11	27	15
US $70,000 to < US $90,000	6	22	12	25
> US $90,000	28	26	18	15
Primary home language (%)				
English	94	89	82	78
Spanish	0	0	6	13
Other	6	11	12	9
*Service utilization in past 6 months*				
Primary caregiver mental health (% yes)	19 +	4 +	24	8
Primary caregiver parenting classes (% yes)	16	15	18	8
Child any services (% yes)	84	93	94	88
Child any ABA (% yes)	55	58	53	54

Children missing either IQ or Vineland data were included in the group with intellectual disability if the non-missing score fell below 76. *T*-scores are standardized scores with a mean of 50 and a standard deviation of 10. Percentages are rounded

+ *p* < 0.10;

* *p* < 0.05

**Table 2:** Attendance and adherence by intervention modality

Variable	MBSR (*n* = 58)			PE (*n* = 59)		
	MBSR In person (*n* = 32)	MBSR Virtual (*n* = 27)	Modality difference	PE In person (*n* = 34)	PE Virtual (*n* = 24)	Modality difference
Attendance						
≥ 1 session (% Yes)	100	89	*χ*^2^(1) = 3.75, *p* = 0.053	97	79	*χ*^2^(1) = 4.86, *p* = 0.028
Majority of sessions (% ≥ 4)	69	92	*χ*^2^(1) = 4.28, *p* = 0.039	82	100	*χ*^2^(1) = 3.91, *p* = 0.048
Total number of sessions	5.5 (2.8)	7.2 (2.3)	*t*(54) = − 2.47, *p* = 0.017	6.3 (2.4)	8.2 (1.1)	*t*(48) = − 3.84, *p* < 0.001
Second caregiver attended (% yes)	38	11	*χ*^2^(1) = 5.38, *p* = 0.020	53	13	*χ*^2^(1) = 9.96, *p* = 0.002
Adherence						
Total homework (weeks attempted or completed)	4.7 (2.3)	5.3 (2.0)	*t*(47) = − 0.966, *p* = 0.339	5.1 (2.3)	5.9 (1.2)	*t*(46) = − 1.77, *p* = 0.083
≥ 4 weeks of homework	75	75	*χ*^2^(1) = 0.00, *p* = 1.000	79	95	*χ*^2^(1) = 2.36, *p* = 0.125

**Table 3:** Results of multilevel models predicting initial status and change in parenting stress over time

		Model 1	Model 2	Model 3
Fixed effects				
Initial status (π_0i_)	Intercept (γ_00_)	− 0.217	− 0.119	0.138
	Intervention group (γ_01_)	0.194	− 0.284	0.294
	Mental health services (γ_02_)	4.653*	5.067**	4.966*
	Months of ABA (γ_03_)	0.365**	0.356***	0.351***
Rate of change (Π_1i_)	Intercept (γ_10_)	− 2.647***	− 2.873***	− 3.257***
	Intervention group (γ_11_)	− 1.970*	− 1.853	− 0.744
	Intervention modality (γ_12_)		0.154	1.365
	Intervention group*modality (γ_13_)			− 3.015
Random effects				
Level 1	Within-person (σ^2^_e_)	36.968	36.600	33.183
Level 2	Initial status (σ^2^_ζ0_)	52.940	49.511	54.098
	Rate of change (σ^2^_ζ1_)	3.919	4.664	5.715
Covariances	(τ_01_)	− 4.538	− 3.840	− 6.307
*R* ^2^	*R* ^2^_*e*_	0.460	0.466	0.516
	*R* ^2^_0_	0.228	0.278	0.211
	*R* ^2^_1_	0.293	0.159	−0.030

Negative *R*^2^ values are not uncommon when running MLMs on longitudinal data. Models 2 and 3 do not fit the data significantly better than Model 1

*ABA*, applied behavior analysis

* *p* < 0.05;

** *p* < 0.01;

*** *p* < 0.001

## Data Availability

Certain project data are available through the National Database for Autism Research (NDAR).
